# Computational Characterization of Small Molecules Binding to the Human XPF Active Site and Virtual Screening to Identify Potential New DNA Repair Inhibitors Targeting the ERCC1-XPF Endonuclease

**DOI:** 10.3390/ijms19051328

**Published:** 2018-04-30

**Authors:** Francesco Gentile, Khaled H. Barakat, Jack A. Tuszynski

**Affiliations:** 1 Department of Physics, University of Alberta, Edmonton, AB T6G 2E1, Canada; jackt@ualberta.ca; 2 Faculty of Pharmacy and Pharmaceutical Sciences, University of Alberta, Edmonton, AB T6G 2H1, Canada; kbarakat@ualberta.ca; 3 Department of Oncology, University of Alberta, Edmonton, AB T6G 1Z2, Canada; 4 Department of Mechanical and Aerospace Engineering, Politecnico di Torino, 10129 Torino, Italy

**Keywords:** DNA repair, ERCC1-XPF, endonuclease, homology modeling, virtual screening

## Abstract

The DNA excision repair protein ERCC-1-DNA repair endonuclease XPF (ERCC1-XPF) is a heterodimeric endonuclease essential for the nucleotide excision repair (NER) DNA repair pathway. Although its activity is required to maintain genome integrity in healthy cells, ERCC1-XPF can counteract the effect of DNA-damaging therapies such as platinum-based chemotherapy in cancer cells. Therefore, a promising approach to enhance the effect of these therapies is to combine their use with small molecules, which can inhibit the repair mechanisms in cancer cells. Currently, there are no structures available for the catalytic site of the human ERCC1-XPF, which performs the metal-mediated cleavage of a DNA damaged strand at 5′. We adopted a homology modeling strategy to build a structural model of the human XPF nuclease domain which contained the active site and to extract dominant conformations of the domain using molecular dynamics simulations followed by clustering of the trajectory. We investigated the binding modes of known small molecule inhibitors targeting the active site to build a pharmacophore model. We then performed a virtual screening of the ZINC Is Not Commercial 15 (ZINC15) database to identify new ERCC1-XPF endonuclease inhibitors. Our work provides structural insights regarding the binding mode of small molecules targeting the ERCC1-XPF active site that can be used to rationally optimize such compounds. We also propose a set of new potential DNA repair inhibitors to be considered for combination cancer therapy strategies.

## 1. Introduction

The human genome is continuously exposed to damage caused by endogenous and exogenous agents. The effects resulting from these lesions range from interfering with cellular processes to inducing mutations which can lead to several pathological conditions. To maintain genome integrity, cells have developed a series of DNA repair pathways, which are able to recognize and repair specific DNA damages through the action of dedicated proteins [1]. In the context of cancer, DNA repair pathways simultaneously can be considered as both friends and enemies. Indeed, although the obvious role of these pathways is to maintain genome stability and remove mutation-causing damages, they can interfere with cancer therapies, which aim to damage the cancer cell genome and hence induce apoptosis. Examples of such therapies are platinum-based chemotherapy and ionizing radiation therapy. Unsurprisingly, the success of these strategies highly depends on the DNA repair capability of the targeted cell population [2,3,4]. Accordingly, a relatively new direction to improve the efficacy of these treatments is to use them in combination with drugs able to inhibit the DNA repair mechanisms [5,6].

Among the five main repair pathways found in humans, the nucleotide excision repair (NER) pathway is dedicated to the repair of bulky DNA lesions which distort the helix structure, interfering with the replication cycle. Such lesions can be caused by ultraviolet light radiation (UV), environmental chemical agents, or reactive oxygen species [1,7]. NER is also responsible for the removal of DNA damages caused by platinum-based chemotherapy drugs such as cisplatin [5]. Over-expression of NER proteins results in cisplatin resistance in cancer cells, whereas cell populations with low-expression of NER proteins are hypersensitive to DNA damaging agents [2,3]. In addition, modulation of NER results in sensitizing cancer cells to DNA-damaging chemotherapy [8].

The NER pathway involves about thirty proteins whose role is to recognize, remove, and replace a damaged DNA strand. One of the essential agents of NER action is the DNA excision repair protein ERCC-1-DNA repair endonuclease XPF (ERCC1-XPF) complex, a 5′-3′ structure-specific endonuclease, which cuts the strand at 5′ of the damaged zone. ERCC1-XPF is also involved in inter-strand crosslink (ICL) and double-strand break (DSB) repair pathways. ERCC1-XPF is a heterodimer composed of two proteins. The first, ERCC1, contains 297 residues divided in a central domain and a double helix-hairpin-helix (HhH2) domain. The second protein, XPF, comprises 916 residues divided in a helicase-like domain; it lacks the helicase activity, a nuclease domain, which contains the catalytic site, and, finally, also contains an HhH2 domain. Dimerization occurs mainly through the two HhH2 domains. An excellent review article about the structure and function of ERCC1-XPF can be found in McNeil et al. [9].

ERCC1-XPF is an attractive target for designing small molecule inhibitors of DNA repair. To inhibit the activity of the ERCC1-XPF endonuclease, three major ways recently have been explored. The first approach is to target the interaction between the central domain of ERCC1 and the DNA repair protein complementing XP-A cells (XPA), through which the endonuclease is recruited to the damage site in NER [10,11]. However, this approach would be effective solely in the inhibition of NER; the ICL and DSB repair activity would be preserved as XPA is not involved in these pathways [9]. A second approach is to target the ERCC1-XPF protein-protein interaction. Our group and others identified and targeted binding pockets at the interface of the dimerized HhH2 domains to inhibit the dimerization of the ERCC1 and XPF, an essential component in the building of a functional endonuclease [12,13]. Although this approach would result in stopping any activity of ERCC1-XPF, it presents some difficulties due to the high-affinity, hydrophobic nature of the involved protein–protein interaction [9]. Finally, the third approach is to target the XPF active site. Recently, McNeil et al. [13], Chapman et al. [14,15], and Arora et al. [16] discovered several small molecule inhibitors targeting the catalytic site of XPF with promising biological activities. The lack of an experimentally determined crystal structure for the XPF nuclease domain as well as the similarity of the active site with related nucleases are the two main drawbacks of this latter approach. Nevertheless, targeting the XPF active site is a promising strategy to inhibit the endonuclease activity as a result of the presence of metal ions in the catalytic site (ideal for metal chelators), the weak contacts established by the domain and the DNA, and the number of successful drug discovery programs targeting DNA repair-related similar enzymes [9].

In this work, we employed computational methods to accomplish the following: (1) build a structural model of the human XPF nuclease domain, which can be used in structure-based drug design and virtual screening (VS); (2) investigate the binding modes of known XPF active site inhibitors, identifying key residues involved in small molecule binding; (3) perform a pharmacophore and structure-based VS campaign against the ZINC Is Not Commercial 15 (ZINC15) compound database [17] to propose potential novel inhibitors binding to the XPF catalytic site. The reported results provide the first detailed investigation of the interactions between the XPF active site and small molecules binding to it. Our findings should be of considerable interest to rationally modify these molecules to improve the binding affinities as well as their specificity to the target. Additionally, we provided a set of commercially available compounds, which can potentially bind to the XPF catalytic site and inhibit the endonuclease activity of ERCC1-XPF; therefore, they can be considered in combination with DNA-damaging cancer therapies to amplify their effects.

## 2. Results and Discussion

### 2.1. Homology Modeling and Molecular Dynamics Simulation

The results obtained from the Molecular Operating Environment (MOE) MOE-SearchPDB protocol are reported in Table 1. We identified top templates based on an expectation value (*E-value*) equal or lower than 1 × 10^12^. Other potential templates with *E-values* between the accepted value and the cutoff were retained only if the associated *Z-scores* were at least 6.

All four of the tested matrixes identified XPF-related proteins from the *Aeropyrum pernix* (PDB ID 2BGW, 2BHN) [18] and *Pyrococcus furiosus* (PDB ID 1J22) [19] archaea. In addition to the hits identified using the Gonnet and Point Accepted Mutation 250 (PAM250) methods, the BLOcks SUbstitution Matrix (BLOSUM) matrices led to the identification of the Mus81 protein (PDB ID 2ZIU (human/*Dario rerio*), 2ZIX (human), and 4P0P (human)) as template as well, which are known to be related to XPF [20]. In contrast to the other three matrices, BLOSUM62 included the Hef protein (1J22) from *Pyrococcus furiosus* within the top templates. Accordingly, we selected the BLOSUM62 results for the successive steps as this matrix showed the best performances in detecting biological relationships, even for distantly related proteins [21,22,23]. The nuclease motif is conserved among XPF family, putative RNA helicases (SF2), and the Mus81 family, and it is represented in human XPF by residues D687, E690, D715, E725, R726, K727, and D731 [24]. In addition to this motif, we observed seven other conserved residues from the multiple sequence alignment, corresponding to V686, L711, G714, S733, G739, Q744, and E760 in the human XPF sequence. The sequence alignments of the XPF nuclease domain and the six templates are reported in Figure S1 in the Supplementary Materials.

**Figure 1:**
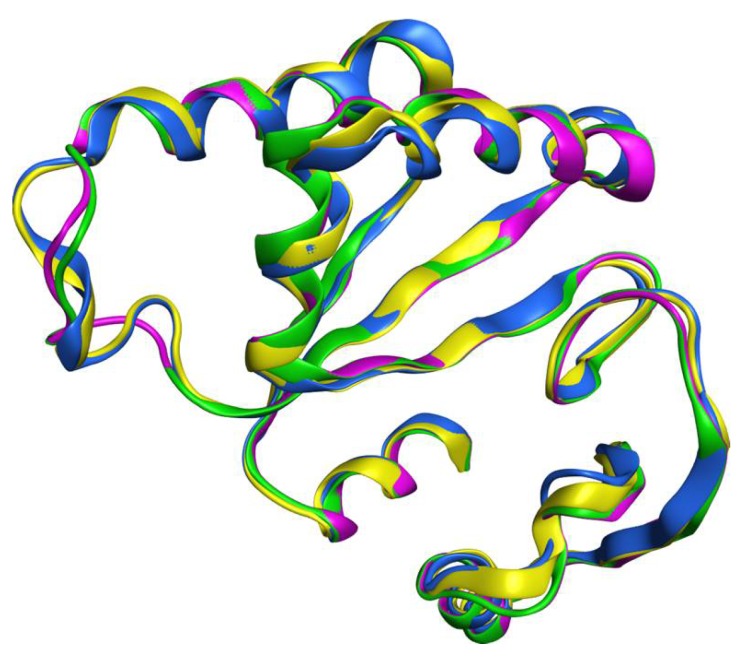
Structural superposition of the four top templates for the human DNA repair endonuclease XPF (XPF) nuclease: 2BGW (blue, XPF from *Aeropyrum pernix*), 2BHN (yellow, XPF from *Aeropyrum pernix*), 1J22 (purple, HeF from *Pyrococcus furiosus*), and 1J25 (green, HeF from *Pyrococcus furiosus*).

The top templates identified by MOE were 2BGW, 2BHN, and 1J22. The metal-binding site of the XPF is likely to employ a two-metal-ion catalysis process to cleave the DNA [25]. However, the available structures contained zero to one metal ion. The absence of a second ion may have been a result of the requirement of a catalytic complex for its stable binding, as in the case of the related Mus81-Eme complex [26]. Also, the majority of known XPF active site inhibitors contain at least one metal-binding motif. For these reasons, we also included the Hef protein from *Pyrococcus furiosus*, which is associated with the PDB ID 1J25; it has the same structure as 1J22 but contains one coordinated metal ion. The four nuclease domains (from 2BGW, 2BHN, 1J22, and 1J25) shared a very similar and superimposable structure (Figure 1). Finally, we selected the nuclease domain of the 1J25 structure as a template with which to build the homology model of the human XPF nuclease domain, based on the highest sequence identity (35.2%) and similarity (60.7%) scores observed among the four sequences and the presence of one metal ion. The alignment of the sequences of the human XPF nuclease domain, 2BGW, and 1J25 is reported in Figure S2 in the Supplementary Materials.

Once the homology model of the human XPF nuclease domain was obtained, we manually modified the Mn^2+^ ion deriving from the HeF structure to a Mg^2+^ ion, which is the biologically relevant cofactor for the ERCC1-XPF endonuclease [9]. The metal ion was stably coordinated by the negatively charged side chains of residues D715 and E725 and the backbone oxygen of R726.

**Figure 2:**
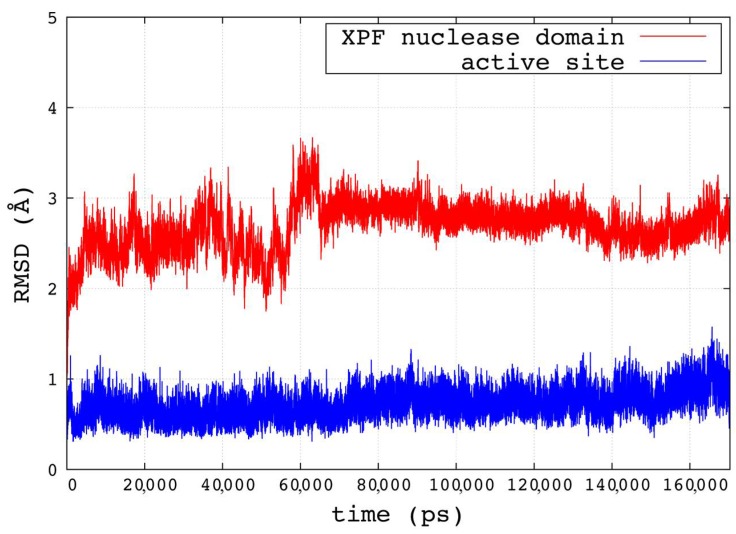
Root-mean-square deviation (RMSD) trends for the backbone atoms of the human XPF nuclease domain (red) and the active site (blue), defined as residues D687, R689, E690, D715, E725, R726, K727, and D731.

The best predicted structure derived from the 1J25 template was simulated with molecular dynamics (MD) for 170 ns. The root-mean-square deviation (RMSD) trend of the backbone atoms of the modeled domain reached a plateau after about 60 ns, with stable fluctuations around 3 Å for the remaining simulation time. The backbone atoms of the active site residues fluctuated steadily around 1 Å for the duration of the simulated time, which followed the restrain release and the initial equilibration phase. The RMSD plots are reported in Figure 2. After visually inspecting the zone surrounding the metal ion during the simulation and considering the previously sequence alignments, we defined the active site as the residues D687, R689, E690, D715, E725, R726, K727, and D731. During the simulation, three stable water molecules completed the coordination of the Mg^2+^ ion (coordination number of six).

A clustering of active site conformations was performed over the last 106 ns of the MD simulation. When cluster counting was equal to 10 clusters, we observed the highest peak of the pseudo-F statistic (pSF) value, a kink in the curve of the ratio between the sum of square regression and the number of total squares (SSR/SST), and a local minimum for the David-Bouldin (DBI) index, indicating optimal cluster counting (see Figure S3 in the Supplementary Materials). Cluster compositions are reported in Table S1 in Supplementary Materials. To exclude rarely occurring active site conformations from the molecular docking simulations, we selected the representative structures from the top six most populated clusters, including 99% of the total conformations, to be used as targets. We also included the lowest potential energy structure (~−98.831 kcal/mol) of the XPF domain extracted by the equilibrated part of the MD simulation.

### 2.2. Modeling of Small Molecules Binding to the Human XPF Active Site

**Figure 3:**
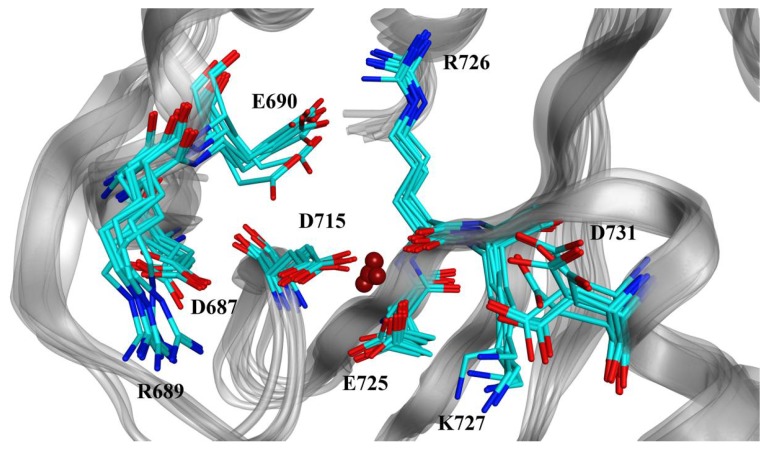
Superposition of the seven conformations of the human XPF active site obtained by clustering the molecular dynamics (MD) trajectory and including the lowest potential energy conformation. The set of conformations was then used as a target for the molecular docking simulations. Carbon, nitrogen and oxygen atoms are represented in cyan, blue and light red, respectively. Dark red spheres indicate the positions of the Mg^2+^ ion present in the active site and coordinated by D715, E725, and R726.

To account for the flexibility of both the side chains and the backbone of the active site, we considered the seven XPF structures described previously as single targets for our docking protocol. A detailed view of these conformations is reported in Figure 3.

**Figure 4:**
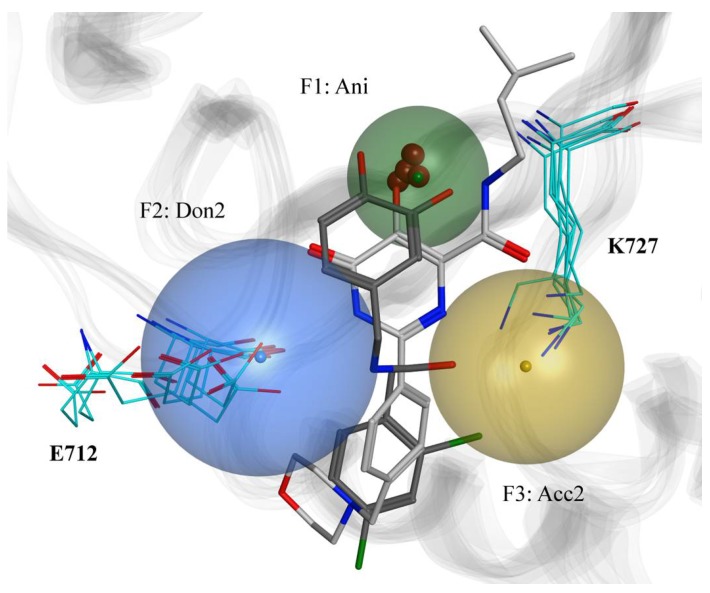
Pharmacophore model designed in consideration of the predicted binding poses of the known XPF inhibitors. Three features were present for all ligand-protein complexes: the anionic one (Ani, green sphere) which was in proximity to the Mg^2+^ ion (dark red spheres), the donor projection (Don2, blue sphere) which was close to E712, and the acceptor projection (Acc2, gold sphere) which was close to K727. Carbon, nitrogen and oxygen atoms of the active site are represented in cyan, blue and light red, respectively. Two docked ligands satisfying the pharmacophore model are also reported, namely **13** [15] (carbon, chlorine, nitrogen and oxygen atoms represented in dark grey, green, blue and light red, respectively), and **33** [14] (carbon, nitrogen and oxygen atoms represented in light grey, blue and light red, respectively). All the conformations of the active site extracted from the clustering of the MD trajectory are represented.

From the resulting binding poses, we identified a pattern of conserved interactions between the small molecules and specific parts of the XPF active site. As expected, the metal-binding motifs present in the ligand structures carried a negative charge and were close to the Mg^2+^ ion. Also, we observed two hydrogen bonds being consistently established between the hydrogen bond donor and acceptor groups of the ligands and E712 and K727, respectively. Therefore, the resulting three-point pharmacophore model included three features: one anionic (Ani) with radius of 2 Å, one donor projection (Don2) with radius of 3.2 Å, and one acceptor projection (Acc2) with radius of 2.7 Å, as represented in Figure 4. It is noteworthy that this pharmacophore model accounted for multiple conformations of the active site, as its design took into consideration that ligands bound to different XPF structures.

### 2.3. Virtual Screening

Approximately 80,600,000 structures from ZINC15 were downloaded. After the filtering step and the pharmacophore-based screening, we reduced the number of compounds to undergo the structure-based VS step to 2,013,120. We then performed VS of the compounds against the set of structures of the XPF nuclease domain and retained only the resulting binding modes which satisfied the pharmacophore features, resulting in retaining only 104,714 unique compounds for consideration. The highest-ranked XPF inhibitor was compound **15**, with a London dG score of −29.543 kcal/mol. 285 hits from the ZINC15 resultant set showed a better score than compound **15**. Visual analysis of the binding modes was then performed to further refine the hit set. Additional details about the resulting top fifty hits, including chemical structures, ZINC IDs, and London dG binding energies are reported in Set S1 in the Supplementary Materials.

**Figure 5:**
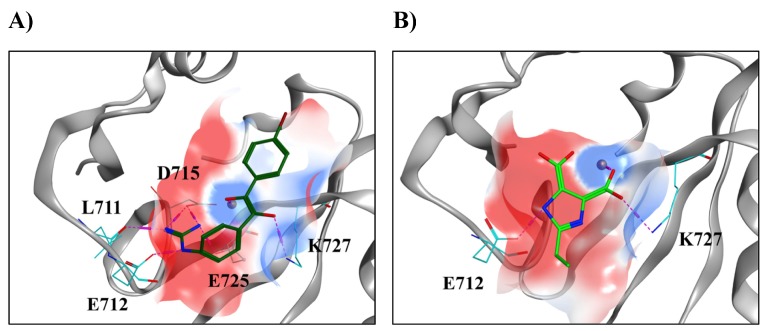
Binding modes of two promising hits derived from the virtual screening (VS). (**A**) ZINC000049131978, hit #5 (carbon, nitrogen and oxygen atoms represented in dark green, blue and light red, respectively). (**B**) ZINC000038550857, hit #13 (carbon, nitrogen and oxygen atoms represented in light green, blue and light red, respectively). Carbon, nitrogen and oxygen atoms of the active site are represented in cyan, blue and light red, respectively. The Mg^2+^ ion is represented as a dark red sphere. Hydrogen bonds are represented in purple dotted lines. The surface of the active site is colored depending on the electrostatic potential, ranging from −40 kcal/mol (intense red) to +40 kcal/mol (intense blue). See text for a detailed description of the electrostatic interactions.

Among the resulting binding modes of the top hits, we observed the dominant interactions were charge-assisted hydrogen bonds between charged groups of the compounds and the charged residues of the XPF active site. The predicted binding modes of two VS-derived hits and examples of non-bonded interactions are reported in Figure 5. Hit #5 (Figure 5A) showed hydrogen bonds between the guanidine group and the side chains of E712 and D715, in addition to a hydrogen bond between the same group and the backbone oxygen of L711. The Mg^2+^ ion interacted with one of the oxygens of the compound, while the other was involved in a hydrogen bond with K727. Hit #13 (Figure 5B), a smaller and less flexible compound, interacted with the Mg^2+^ ion and K727 via one of its carboxyl groups as well as with E712 through a hydrogen bond with the imidazole ring.

On the basis of the binding poses of our predicted hits, we were able to identify in detail the electrostatic features of the active site which are important for ligand binding. The active site of the human XPF is divided between two zones with different electrostatic properties. The first is a negatively charged part constituted by acidic residues such as E712, D715, and E725 (in red in Figure 5), favorable in the establishment of interactions with electropositive moieties of the ligands. The second is a positively charged part constituted by the metal ion and K727, favorable for interactions with electronegative moieties of the ligands.

## 3. Materials and Methods

### 3.1. Homology Modeling of the Human XPF Nuclease Domain

The amino acid sequence of the human XPF nuclease domain was defined as residue 658 to 813 according to the entry Q92889 in the UniProt database [27]. We used the MOE 2013 (Chemical Computing Group, Montreal, QC, Canada) package for the entire homology modeling process [28]. Initially, we used the MOE-SearchPDB module [29] to align the target sequence with a database of pre-clustered families of proteins [30] for which experimental structures are available in the Protein Data Bank (PDB) [31]. In this way, potential template structures could be identified for use in homology modeling. The parameters for the homology search were chosen as follows: a gap start penalty of −12, a gap extend penalty of −2, an *E-value* cutoff of 10, an *E-value* acceptance of 1 × 10^12^, 100 Z-iterations and a *Z-score* cutoff of 6. As a substitution matrix, we tested the BLOSUM62, BLOSUM50 [32], Gonnet [33], and PAM250 [34], all of which are available in MOE 2013. MOE-Align [29], using sequence and structural alignment, was used for multiple alignment in the following ways. First, the entire XPF sequence was aligned to the identified templates. Second, the XPF nuclease sequence was aligned to the first multiple alignment to obtain a better alignment of the nuclease domains of the templates. Just the nuclease domain sequences were used in successive steps, a trim of the templates’ sequences to the residues aligned within residues 658 and 813 of the human XPF nuclease domain. Accordingly, the best template obtained from this step was used for the homology model building. The parameters were set at 10 intermediate models, one side chain model for each intermediate at 300 K, medium refinement for intermediates, and the Generalized Born/volume integral (GB/VI) [35] scoring for the selection of the final model. The final refinement was set to “Fine” with a root-mean square (RMS) gradient of 0.1 kcal/mol, and the protonation states of the final model were assigned using Protonate3D [36]. Amber ff12SB force field [37] was selected for the entire process. Coordinated metal ions present in the template were included in the process as the environment for the induced fit.

### 3.2. Molecular Dynamics Simulation and Clustering of the Trajectory

Amber ff14SB force field parameters were assigned to the protein [38], whereas the Li, Song, and Merz’s 12-6-4 parameters for mono and divalent ions in TIP3P water were assigned to the ions [39,40]. The protein was solvated with an octahedral box of TIP3P explicit water molecules with 15 Å of minimal distance between the protein atoms and the box edges. Na^+^ and Cl^−^ ions were added to neutralize the system and to simulate a physiological ionic concentration of 0.15 M. The system was simulated in Amber pmemd.cuda [41,42] using the following protocol: relaxation of the NaCl ions and water molecules using 1000 steps of steepest descent and 1000 steps of conjugate gradients minimization, which kept the entire protein and the metal co-factor harmonically restrained (force constant of 500 kcal/mol/Å^2^). 2000 steps of steepest descent were followed by 3000 of conjugate gradients method for the whole system. Subsequently, we performed gradual heating of the system from 0 to 300 K in 100 ps using the Langevin thermostat, keeping the backbone atoms and the co-factor restrained (force constant of 2 kcal/mol/Å^2^) and using an integration time step of 0.5 fs and periodic volume conditions. Gradual release of the restraints followed from 2 to 0 kcal/mol/Å^2^ in four phases of 50 ps each at constant pressure (1 atm), using an integration time step of 2 fs. We then ran 170 ns of production simulation in isothermal-isobaric conditions (NPT), recording the atomic coordinates every 2 ps. The SHAKE algorithm was used to keep the bonds involving hydrogens frozen [43]. The cutoff for long-range interactions was set to 9 Å. To assess the equilibration of the system, we evaluated the time evolution of the mass-weighted RMSD, which was calculated over the backbone atoms of the protein and the active site using cpptraj from AmberTools12 [37]. In addition, the trajectory was visually analyzed using Visual Molecular Dynamics (VMD) program [44]. To extract a set of representative and diverse conformations of the active site to be used as a relaxed complex scheme (RCS) docking protocol [45], we performed RMSD-based clustering of the last 106 ns of the simulation, using the conformations extracted every 10 ps. Firstly, all the translation and rotation motions were eliminated by RMS-fitting the backbone atoms’ positions of the trajectory to the first frame. The average linkage clustering algorithm, as implemented in cpptraj, was then used to divide the frames into clusters and to extract the centroid or representative conformation of each one on the basis of the positions of all the atoms of the active site. In general, the optimal number of clusters is not known a priori. To identify the optimal number of clusters in which the trajectory should be divided, we varied it from 1 to 200 and evaluated three metrics each time, namely the DBI, pSF, and the SSR/SST. A local minimum of the DBI, a maximum of the pSF, and a kink in the SSR/SST plot are expected at the optimal cluster counting [10,46,47].

### 3.3. Molecular Docking of Known Inhibitors and Pharmacophore Modeling

**Figure 6:**
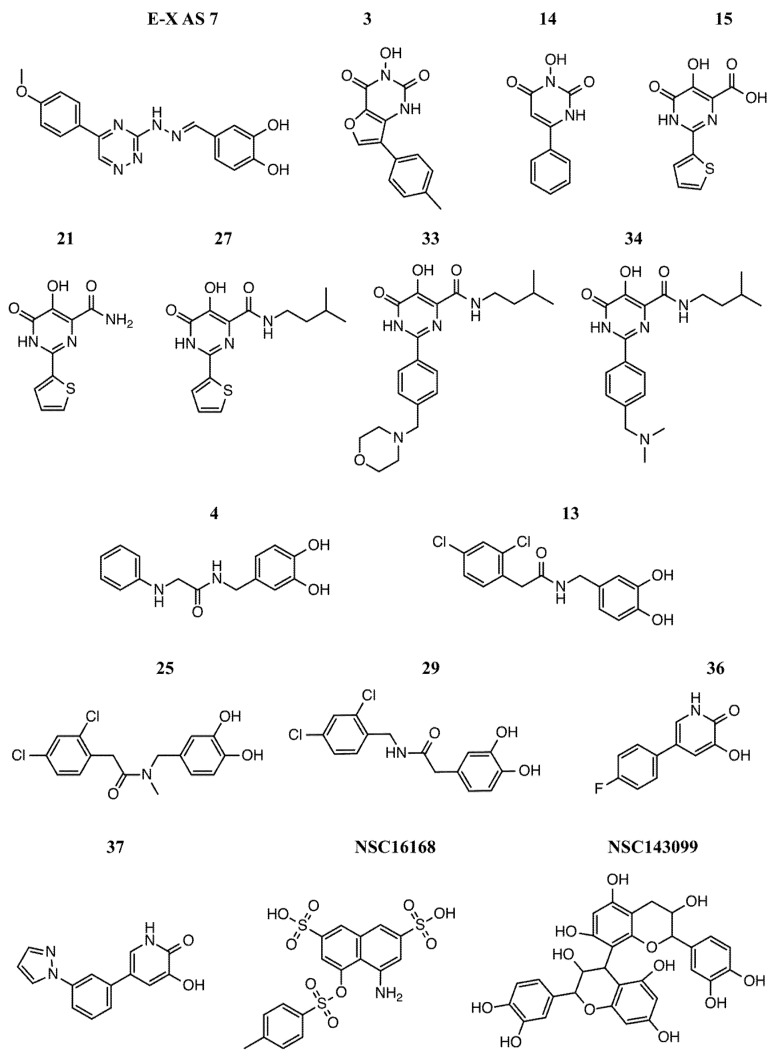
Chemical structures of the XPF inhibitors used to build the pharmacophore model after molecular docking simulations targeting the human XPF nuclease domain were performed. All the small molecules investigated in this step included at least one metal-binding motif.

Molecular docking simulations were run for ERCC1-XPF endonuclease small molecule inhibitors which were likely to bind to the XPF active site: compounds E-X AS7 from McNeil et al. [13], 3, 14, 15, 21, 27, 33 and 34 from Chapman et al. [14], 4, 13, 25, 29, 36, 37 from Chapman et al. [15], and NSC16168 and NSC143099 from Arora et al. [16]. The selection criteria used to select these compounds were the high potencies as ERCC1-XPF activity inhibitors and the specificities to the target. Specifically, we aimed to identify the intramolecular interactions which were essential for a strong and specific binding to the XPF active site. Different accessible protonation states and tautomeric forms of the compounds were obtained using the MOE Database Wash tool. The chemical structures of the small molecules are reported in Figure 6.

We extracted the centroid conformations of the top six clusters found by clustering the MD trajectory to use them as target structures for the docking. In this set, we also included the lowest potential energy protein conformation found in the equilibrated fraction of the MD trajectory. Because all the selected active site inhibitors contained a metal-binding motif in their structure, we used MOE Site Finder to identify a potential binding zone in each structure. This was accomplished by selecting each time the highest ranked site was close to the metal ion. For the docking simulations, we used the Triangle Matcher placement algorithm [48], which returned thirty poses; we also used the Rigid Receptor refinement method which returned one final pose, as implemented in MOE Dock. The London dG method [35,49] was used to score the poses in both steps. The binding energy of a ligand-receptor complex was calculated with the London dG method as

(1) $$\Delta G_{LdG}=c+E_{flex}+\underset{hbonds}{\sum }c_{hb}f_{hb}+\underset{metal-lig}{\sum }c_{m}f_{m}+\underset{i}{\sum }\Delta D_{i}$$

where c is an empirically derived term modeling the change in rotational/translational entropy upon binding; $c_{hb}$ and $c_{m}$ are the energies of ideal hydrogen bonds and metal ligations, respectively; $f_{hb}$ and $f_{m}$ range between 0 and 1 and measure the geometric imperfections of hydrogen bonds and metal ligations, respectively; and $\Delta D_{i}$ is the desolvation contribution modeled by using a volume integral London dispersion [35]. Pharmacophore features common to all the docked compounds were automatically generated using the Consensus method in the Pharmacophore Editor in MOE and the Unified pharmacophore scheme [50].

### 3.4. Virtual Screening of the ZINC15 Database

To identify potential novel ERCC1-XPF inhibitors acting by binding to the XPF active site, we performed a VS campaign against the ZINC1 database, containing ~120 millions of compounds. Prior to performing the VS, the database needed to undergo several filtering and preparation steps to reduce the enormous number of compounds and, at the same time, to consider different states of the retained small molecules. Hence, we downloaded all the compounds which satisfied the following ZINC15 filters: availability of 3D conformers, standard reactivity, commercial purchasability as wait-ok, standard protonation state at pH of 7.4, and charges ranging from −2 to +2. We then used the MOE Database Wash tool again to calculate other accessible protonation states and tautomers. We filtered the resulting database to retain just the compounds satisfying the Oprea’s lead-like filter [51]. The Oprea’s rules are (a) the number of N or O atoms that are hydrogen bond donors must be maximum 5; (b) the number of N and O atoms must be maximum 8; (c) the molecular weight must be maximum 450; (d) the logP must be between −3.5 and 4.5; (e) the number of rings of size three through eight must be maximum 4; and, (f) the number of rotatable bonds must be maximum 10. A compound is considered lead-like if its structure violates, at most, one of Oprea’s rules. Our goal was to screen only lead-like compounds which could be optimized in drug-like compounds once the activities were assessed. As the last preparation step, we generated up to five 3D conformations for each compound in MOE, imposing a strain limit of 4 kcal/mol. The pharmacophore obtained previously was then used to screen the resulting multi-conformational database, retaining only those compounds with at least one conformation satisfying the pharmacophore. We then performed a structure-based VS of the resulting ZINC15 subset of small molecules, using the same docking parameters described previously. The resulting top-scored poses were filtered again using the same pharmacophore model to retain only the binding modes which satisfied the pharmacophore features. Duplicates of the same compound scoring worse than the top pose were removed.

## 4. Conclusions

The ERCC1-XPF endonuclease plays a primary role in several DNA repair pathways, including NER, ICL, and DSB. Because these pathways in cancer cells are involved in the repair of damages caused by DNA-damaging cancer therapies, blocking their activity was expected to result in the enhancement of the effect of such therapies. An inhibition of endonuclease activity through the use of small molecules binding to the catalytic site of XPF is a relatively new strategy, which has not yet been fully explored. Indeed, despite the recent discovery of several XPF active site inhibitors, structural information is lacking regarding the mode of binding of these compounds, largely a result of the unavailability of experimental structures of the human XPF nuclease domain. Here, we generated a homology model for such a domain, based on templates that were carefully selected among all the structures of protein domains related to that of the human XPF. Consequently, we used MD simulations and iterative clustering of the MD trajectory to identify dominant conformations of the active site and used the resulting set of structures as targets in molecular docking simulations of the most potent and selectively known XPF inhibitors. As a result, we built a pharmacophore model elucidating the key interactions required for an effective ligand binding to the site, involving E712 and K727 as well as the coordinated Mg^2+^ ion. A multi-step VS campaign was then performed to identify potential novel XPF inhibitors by sequentially filtering the ZINC15 database.

This work provides a detailed picture of the binding modes of small molecules to the human XPF active site. The results presented here can be effectively used in the rational design of XPF inhibitors, which are potent and specific to the target. In addition, we identified a set of commercially available chemical compounds which can potentially show improved binding compared to the set of known inhibitors. To validate our model of ligand binding, mutation studies regarding the residues identified as essential for binding would be ideal. On the basis of previous studies, residue E712 would be the best candidate for a mutation study, as it is not conserved among other XPF-related proteins and its mutation does not affect the endonuclease activity. On the other hand, K727 would stop the activity when mutated [24]. Additionally, the top hits we reported in this study represent a good starting point to rationally develop optimized analogues following their experimental validation as XPF binders and DNA repair inhibitors.

## Figures and Tables

**Table 1:** Results from different substitution matrices available in MOE 2013 for the detection of homologs of the human XPF nuclease domain. Proteins are reported with their PDB ID. See text for more details.

Substitution Matrix	Top Templates	Others
BLOSUM50	2BGW, 2BHN	1J22, 2ZIU, 4P0P
BLOSUM62	2BGW, 2BHN, 1J22	2ZIU, 2ZIX, 4P0P
Gonnet	2BGW, 2BHN	1J22
PAM250	2BGW, 2BHN	1J22

## Supplementary Materials

Supplementary materials can be found at http://www.mdpi.com/1422-0067/19/5/1328/s1.
