# Supplementary material for: Computational Characterization of Small Molecules Binding to the Human XPF Active Site and Virtual Screening to Identify Potential New DNA Repair Inhibitors Targeting the ERCC1-XPF Endonuclease
Source: Int J Mol Sci. 2018 Apr 30;19(5):1328. doi: 10.3390/ijms19051328 (PMC5983712; doi:10.3390/ijms19051328)
Supplement: Supplementary file 1 [file ijms-19-01328-s001.zip › SM/ijms-299420-Set S1-pr.pdf]

1

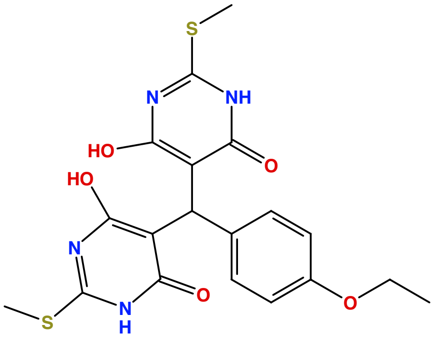

ZINC000008716670

S: -37.4250

2

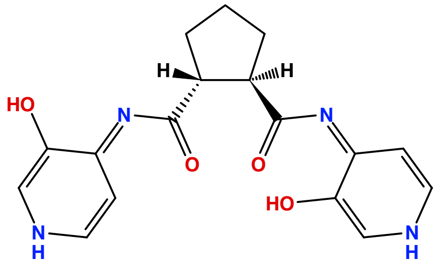

ZINC000343571284

S: -36.8954

3

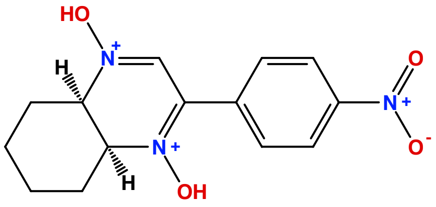

ZINC000225405110

S: -36.3228

4

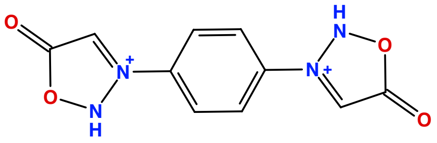

ZINC000006536259

S: -35.9038

5

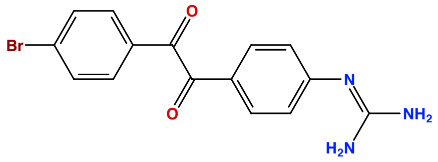

ZINC000049131978

S: -35.3001

6

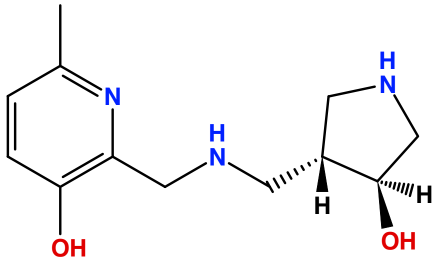

ZINC000262069698

S: -34.4474

7

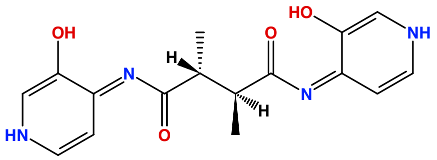

ZINC000358583786

S: -34.4412

8

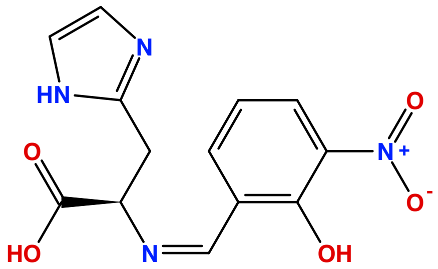

ZINC000104212093

S: -34.0721

9

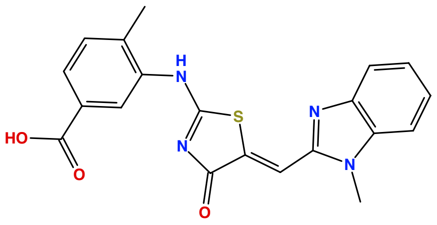

ZINC000020227150

S: -33.6701

10

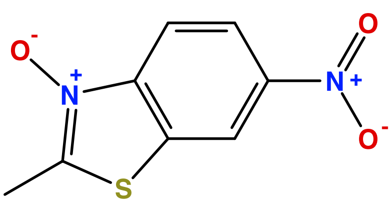

ZINC000045237915

S: -33.6330

11

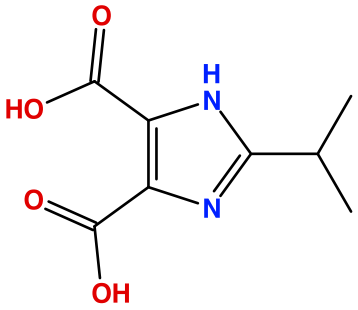

ZINC000071774011

S: -33.5102

12

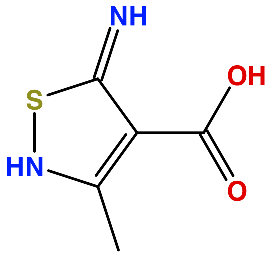

ZINC000032714687

S: -33.0514

13

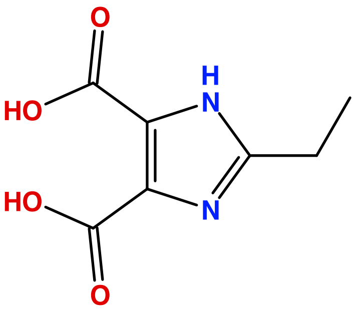

ZINC000038550857

S: -32.8777

14

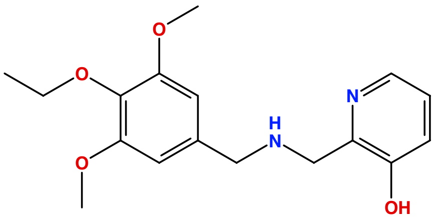

ZINC000347902752

S: -32.8049

15

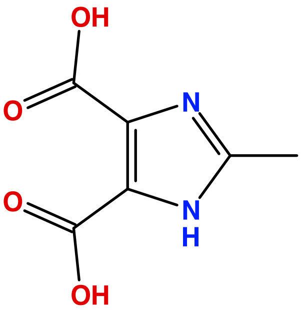

ZINC000015886568

S: -32.7778

16

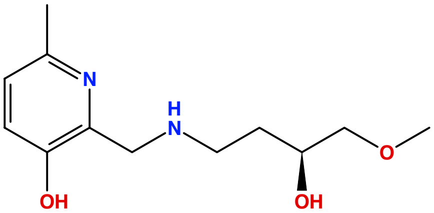

ZINC000231608350

S: -32.3570

17

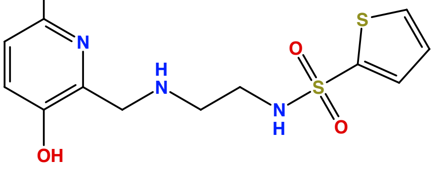

ZINC000122283098

S: -32.3530

18

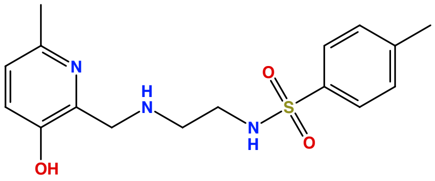

ZINC000103344220

S: -32.3287

19

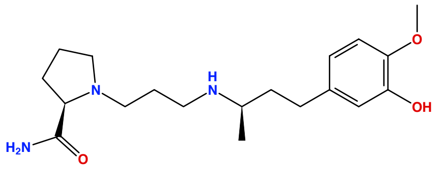

ZINC000277276522

S: -32.2163

20

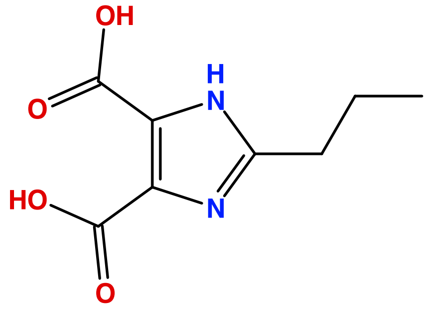

ZINC000021299679

S: -32.1401

21

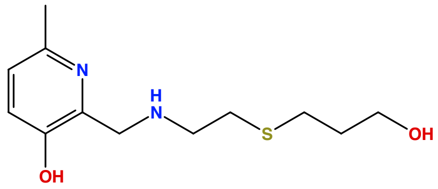

ZINC000105643133

S: -32.1053

22

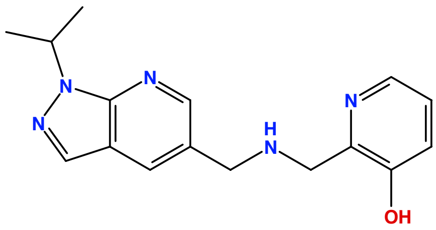

ZINC000348012855

S: -32.0948

23

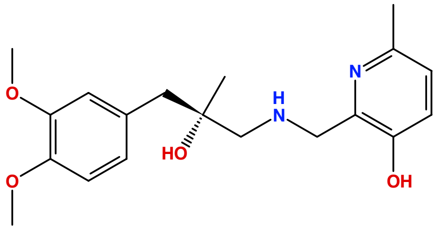

ZINC000289920009

S: -31.9850

24

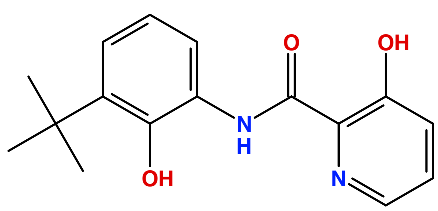

ZINC000361793164

S: -31.9330

25

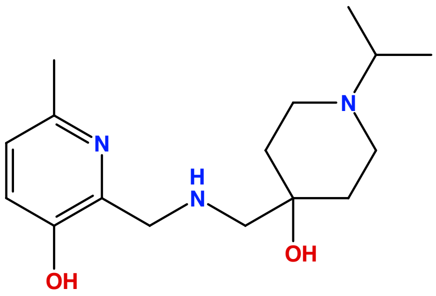

ZINC000268407008

S: -31.9033

26

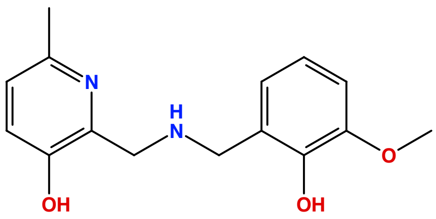

ZINC000265089061

S: -31.7699

27

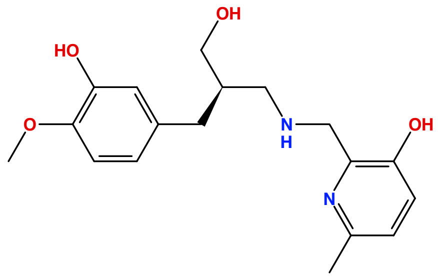

ZINC000289505839

S: -31.7633

28

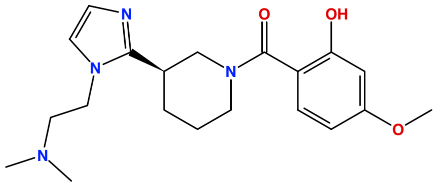

ZINC000095501050

S: -31.7463

29

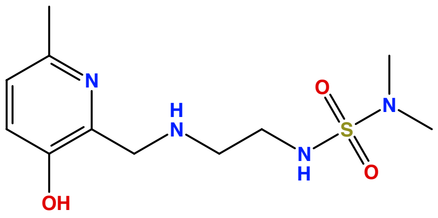

ZINC000291399311

S: -31.7250

30

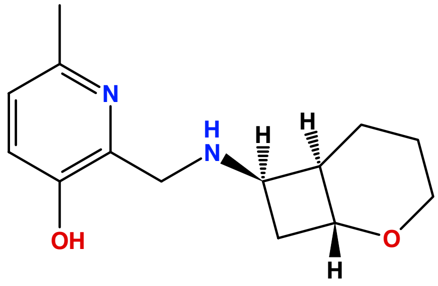

ZINC000365283038

S: -31.6944

31

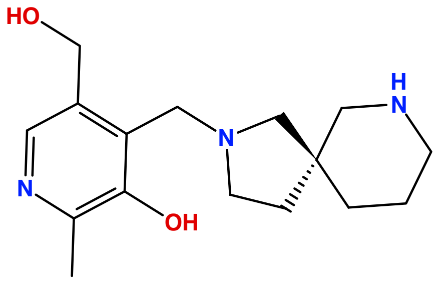

ZINC000067895942

S: -31.6731

32

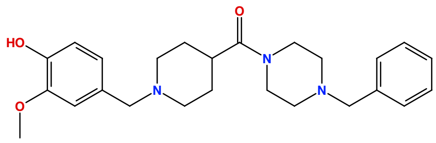

ZINC000000819207

S: -31.5902

33

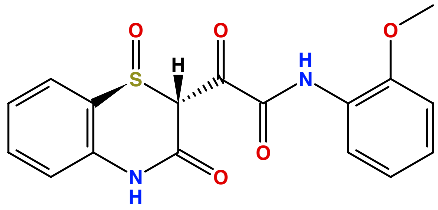

ZINC000005824033

S: -31.5793

34

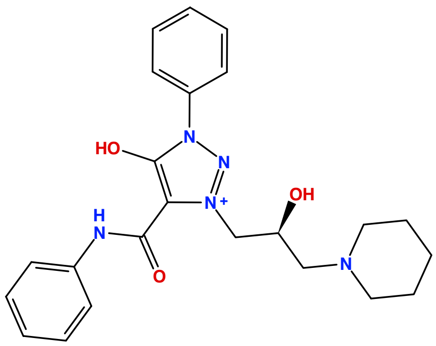

ZINC000025182949

S: -31.5429

35

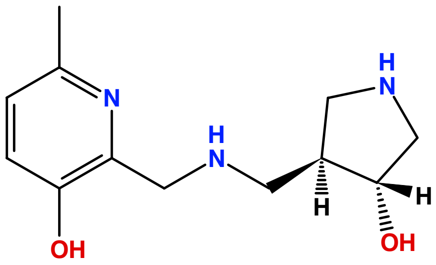

ZINC000262069699

S: -31.4854

36

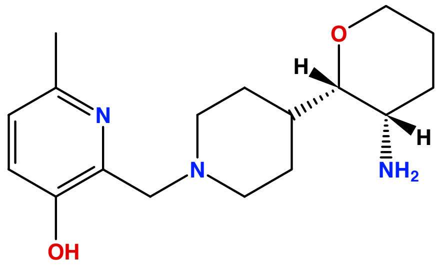

ZINC000333182225

S: -31.4640

37

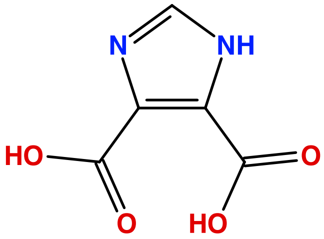

ZINC000015852947

S: -31.4342

38

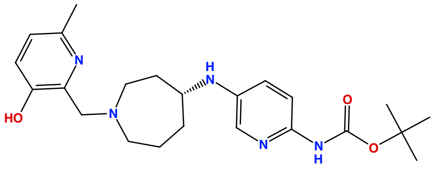

ZINC000170618062

S: -31.3782

39

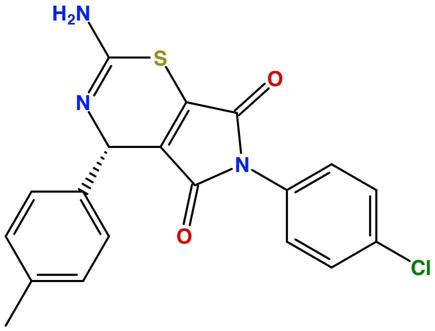

ZINC000002219244

S: -31.3611

40

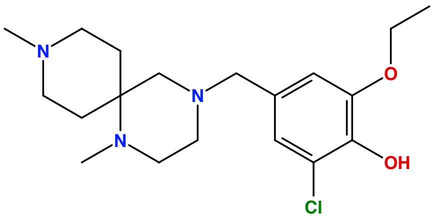

ZINC000067886560

S: -31.3346

41

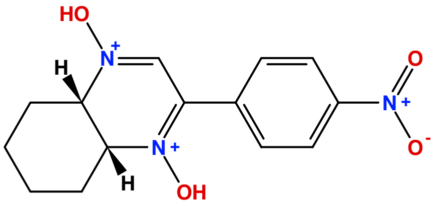

ZINC000225405065

S: -31.2085

42

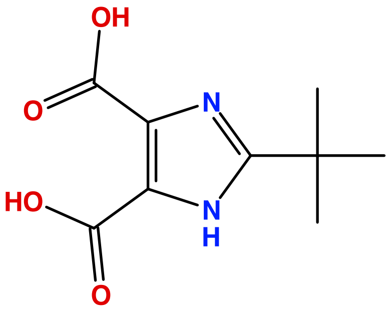

ZINC000038550858

S: -31.2061

43

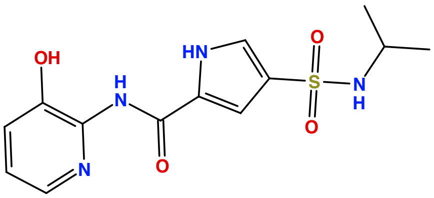

ZINC000341887368

S: -31.1700

44

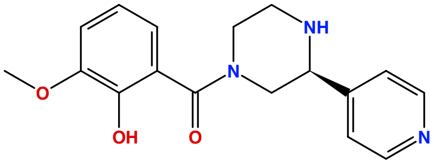

ZINC000374391981

S: -31.1451

45

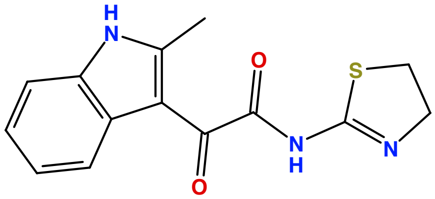

ZINC000030601464

S: -31.0931

46

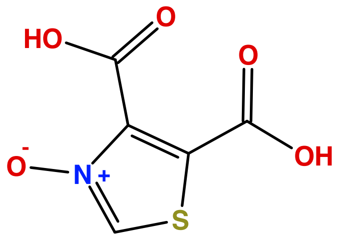

ZINC000247390923

S: -30.9737

47

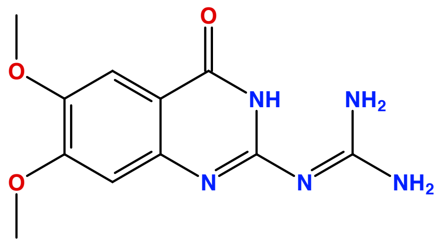

ZINC000006136419

S: -30.8818

48

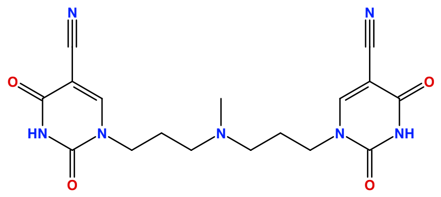

ZINC000008431352

S: -30.8005

49

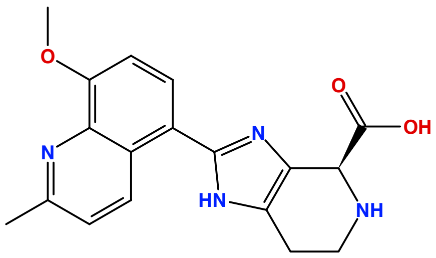

ZINC000077503807

S: -30.7688

50

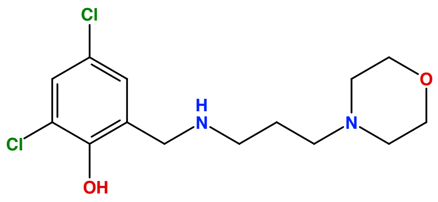

ZINC000021519793

S: -30.6758
