# Supplementary material for: Computational Characterization of Small Molecules Binding to the Human XPF Active Site and Virtual Screening to Identify Potential New DNA Repair Inhibitors Targeting the ERCC1-XPF Endonuclease
Source: Int J Mol Sci. 2018 Apr 30;19(5):1328. doi: 10.3390/ijms19051328 (PMC5983712; doi:10.3390/ijms19051328)
Supplement: Supplementary file 1 [file ijms-19-01328-s001.zip › SM/ijms-299420-Supplementary Figures-pr.docx]

**Supplementary Materials**


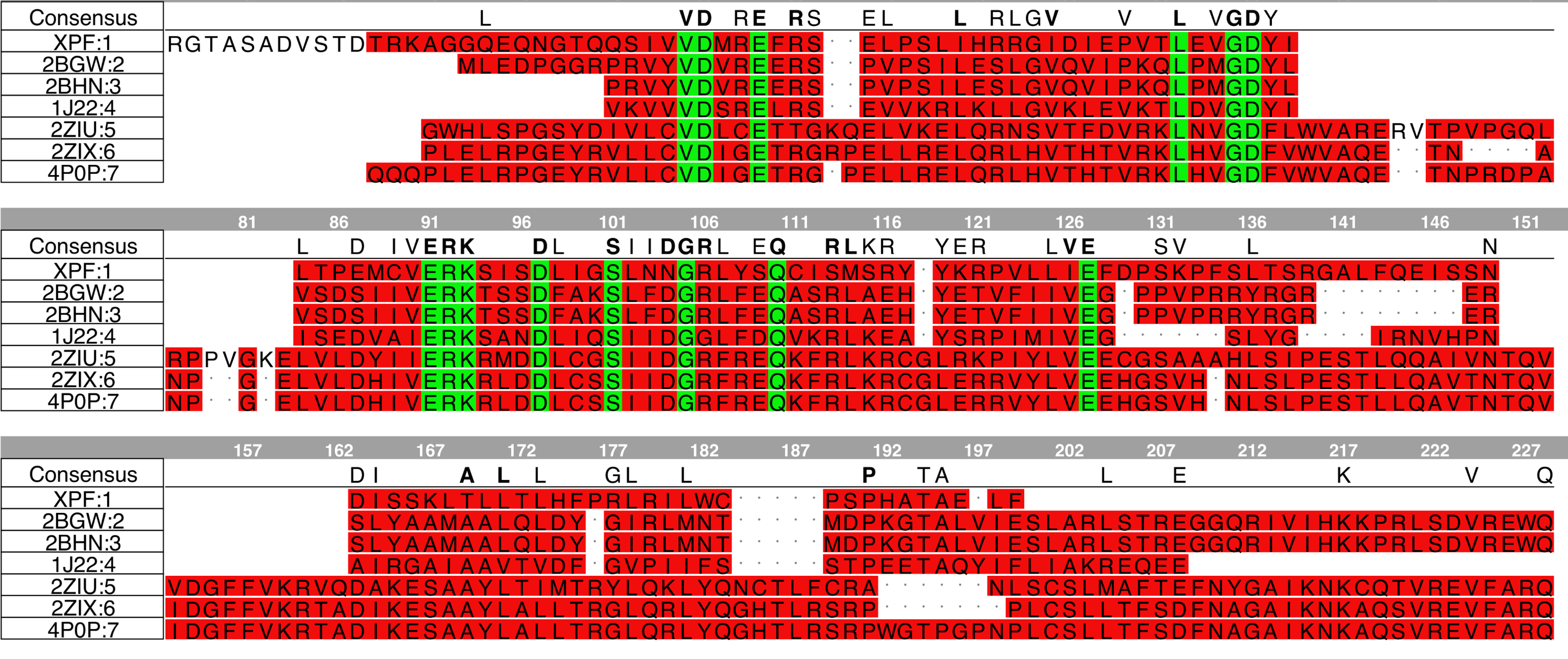
**Figure S1.** Alignment of the human XPF nuclease domain sequence and all the sequences identified as templates by the template search step in MOE, using the BLOSUM62 matrix. Conserved residues are highlighted in green.

**
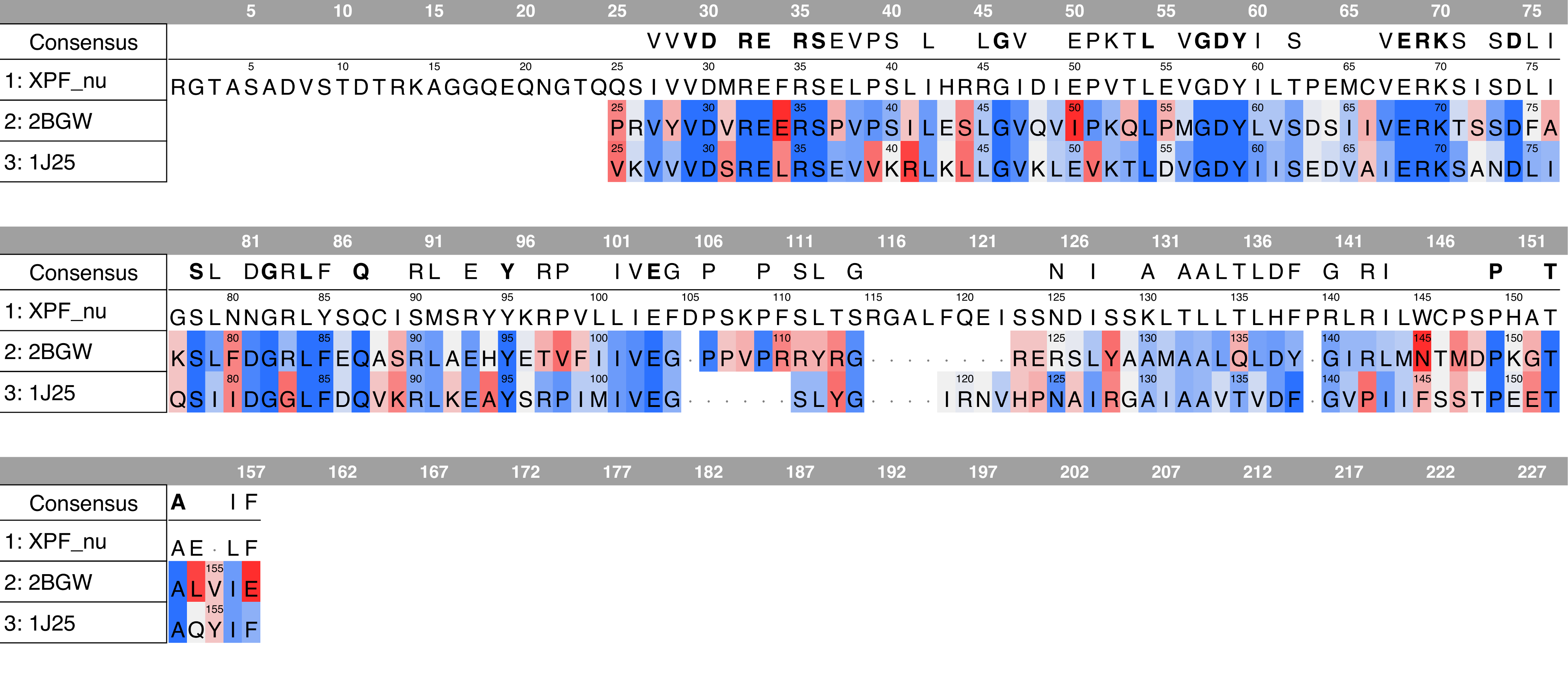
Figure S2.** Alignment of the amino acid sequences of the human XPF nuclease domain, 2BGW and 1J25. Fully conserved residues are reported in the Consensus line in bold. Partially conserved residues are reported in the Consensus line in normal font. Residue columns are colored based on their degree of conservation when compared with the XPF nuclease sequence, ranging from red (not conserved) to blue (fully conserved).


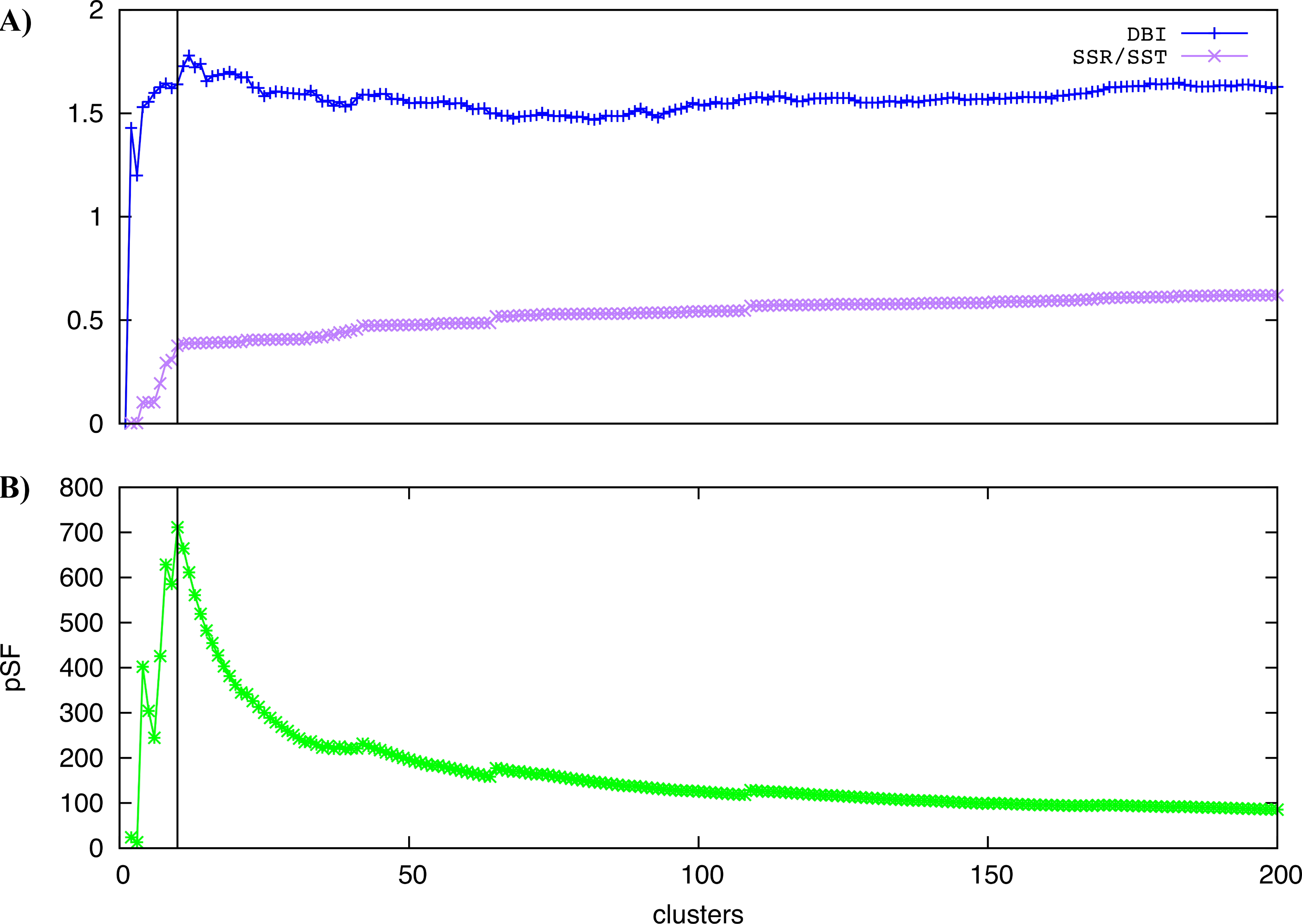


**Figure S3.** A) DBI (blue) and SSR/SST ratio (purple) and B) pSF (green) trends obtained by varying the number of clusters of human XPF active site conformations. At 10 clusters (black vertical line), we observed a local minimum of the DBI, a kink in the SSR/SST curve and a maximum of the pSF, indicating optimal cluster counting.

**Table S1.** Composition of the 10 clusters resulting from clustering of the last 106 ns of MD trajectory. The ratio is calculated as number of frames in the cluster divided by the total number of frames (10601). Selected refers to the representative structure of a cluster being included in the molecular docking simulation as target or not.

| **Cluster** | **Number of frames** | **Ratio** | **Selected** |
| --- | --- | --- | --- |
| 1 | 4160 | 0.392 | yes |
| 2 | 2125 | 0.200 | yes |
| 3 | 5 | 0.000 | no |
| 4 | 1 | 0.000 | no |
| 5 | 1370 | 0.129 | yes |
| 6 | 156 | 0.015 | yes |
| 7 | 15 | 0.001 | no |
| 8 | 1496 | 0.141 | yes |
| 9 | 1264 | 0.119 | yes |
| 10 | 9 | 0.001 | no |
